# Feelings of Disgust and Disgust-Induced Avoidance Weaken following Induced Sexual Arousal in Women

**DOI:** 10.1371/journal.pone.0044111

**Published:** 2012-09-12

**Authors:** Charmaine Borg, Peter J. de Jong

**Affiliations:** Department of Clinical Psychology and Experimental Psychopathology, University of Groningen, Groningen, The Netherlands; Catholic University of Sacred Heart of Rome, Italy

## Abstract

**Background:**

Sex and disgust are basic, evolutionary relevant functions that are often construed as paradoxical. In general the stimuli involved in sexual encounters are, at least out of context strongly perceived to hold high disgust qualities. Saliva, sweat, semen and body odours are among the strongest disgust elicitors. This results in the intriguing question of how people succeed in having pleasurable sex at all. One possible explanation could be that sexual engagement temporarily reduces the disgust eliciting properties of particular stimuli or that sexual engagement might weaken the hesitation to actually approach these stimuli.

**Methodology:**

Participants were healthy women (*n* = 90) randomly allocated to one of three groups: the sexual arousal, the non-sexual positive arousal, or the neutral control group. Film clips were used to elicit the relevant mood state. Participants engaged in 16 behavioural tasks, involving sex related (e.g., lubricate the vibrator*)* and non-sex related (e.g., take a sip of juice with a large insect in the cup) stimuli, to measure the impact of sexual arousal on feelings of disgust and actual avoidance behaviour.

**Principal Findings:**

The sexual arousal group rated the sex related stimuli as less disgusting compared to the other groups. A similar tendency was evident for the non-sex disgusting stimuli. For both the sex and non-sex related behavioural tasks the sexual arousal group showed less avoidance behaviour (i.e., they conducted the highest percentage of tasks compared to the other groups).

**Significance:**

This study has investigated how sexual arousal interplays with disgust and disgust eliciting properties in women, and has demonstrated that this relationship goes beyond subjective report by affecting the actual approach to disgusting stimuli. Hence, this could explain how we still manage to engage in pleasurable sexual activity. Moreover, these findings suggest that low sexual arousal might be a key feature in the maintenance of particular sexual dysfunctions.

## Introduction

*“A man, who will kiss a pretty girl’s mouth passionately, may perhaps be disgusted by the idea of using her tooth-brush.”* Sigmund Freud.

Sex as a procreation stance and disgust as a defensive mechanism, are both basic, evolutionary relevant functions, yet their relationship is paradoxical and possibly obstructive. Disgust has been argued to be evolved as a defensive mechanism to protect the organism from external contamination [1], [2]. Consequently, the main organs or body parts that are involved in this defensive mechanism are known to lie on the border of the body. Accordingly, the mouth and vagina are amongst the body parts that show strongest disgust sensitivity, possibly due to their aperture and higher perceived risk of contamination [3]. In addition, the stimuli involved in sexual encounters are in general (at least out of context) strongly perceived to hold high disgust qualities, with saliva, sweat, semen and body odours qualifying among the strongest disgust elicitors [3]. Clearly then, disgust may be an important interfering factor in sexual activity which may help to explain the mechanisms involved in sexual dysfunction [4], [5].

The finding that many of the strongest disgust eliciting stimuli are also involved in sex (e.g., saliva, and sweat) may not only help explain how disgust may be involved in sexual dysfunction, but it also raises the critical question of how people succeed in having pleasurable sex at all. One possible explanation could be that sexual engagement temporarily reduces the disgust eliciting properties of particular stimuli. Another hypothesis could be that sexual engagement might weaken the hesitation to approach disgust eliciting stimuli. Consequently, this would motivate further approach behaviour, in spite of the unchanged disgust properties of the stimuli. Alternatively, both mechanisms could act in concert. In line with the above, another possible explanation is that the disgust properties of specific stimuli might more readily decrease (i.e., habituate), when being sexually aroused during actual exposure to these disgusting stimuli.

Germane to this, a recent experimental study investigated whether sexual arousal may indeed reduce the disgust properties of specific stimuli in male participants. To elicit sexual arousal, the experimental group watched erotic female images. These male students were then exposed to a series of sex related and non-sex related disgust elicitors that were drawn from various sensory modalities (i.e., visual, tactile, auditory, and olfactory). For example as tactile disgust elicitors, participants were asked to place their dominant hand through a small opening (so the content was not visible) in a bucket containing either four lubricated condoms (sex related) or cold pea and ham soup (non-sex related) while their nostrils were blocked with cotton wool plugs to prevent the perception of any relevant smells. Interestingly, participants in the experimental group subjectively reported being less disgusted by sex related disgust elicitors than participants in the control conditions who were not sexually aroused [6]. Consistent with this, a correlational study showed that both men and women reported less disgust after watching an erotic film when they were more sexually aroused [7]. Similarly, other studies have shown that sexual motivation can distort judgements about the risk of contracting sexually transmitted disease, and sexual arousal has been shown to have a strong impact on decision making [8]. In a similar vein it has been demonstrated that men when sexually aroused reported that they would consider having sex with a woman who is extremely fat, which contrasted their perceptions and reported repulsion when they were not sexually engaged [9]. Therefore one can argue that sexual arousal may attenuate all kinds of mechanisms that may act in a way to avoid particular sexual behaviours or stimuli - be it general repulsion, moral borders (e.g., having sex with a 12 year old) or contamination risk (e.g., condom use). Thus, sexual arousal may influence mechanisms that normally help people avoid certain (disgusting) stimuli.

Although previous findings seem to partially elucidate why people still approach particular stimuli and engage in sex, thus far these findings are restricted to subjective feelings or self-report measures about imagined situations [6]–[9]. It would therefore be important to further investigate whether experimentally induced sexual arousal is not only successful in reducing deliberately reported disgust but also people’s willingness to actually approach particular initially disgusting stimuli. The avoidance response is significant because disgust may create distance from the disgusting stimuli and thus interfere with sexual behaviours. It could very well be that behaviour is modulated by sexual arousal and consequently weakens the tendency to avoid. For instance, a reduction of subjective disgust in the condition of sex or a sexual encounter could follow merely by being in contact with a particular stimulus. Besides, these earlier findings on the impact of sexual arousal on the disgust-eliciting properties of particular sexual stimuli were predominantly restricted to men [6]. Given the evolutionary differential roles of men and women, women’s higher sensitivity to disgust [10], [11] and their higher vulnerability to infections [12], it would be interesting to investigate whether these findings are also robust in a female sample. Therefore, the present study was designed to test whether in women also a sexual arousal induction would attenuate disgust in response to sex related disgusting stimuli. Importantly, we not only examined the influence of sexual arousal on the subjective feelings of disgust but also tested whether sexual arousal would facilitate participants’ actual approach towards disgusting stimuli. Moreover, in order to test whether this reduction in disgust properties would be restricted to sexual stimuli or would represent a more general phenomenon that applies to disgusting stimuli in general, we also included generally disgusting stimuli that do not directly refer to sex (i.e., non-sex related).

In addition, previous evidence suggested that disgust is not a unitary emotion but that there are different subtypes. Current research suggest that four different categories of disgusting stimuli can be differentiated, namely core, animal-reminder, contamination and moral disgust stimuli [2], [13]. It has been argued that disgust originated from oral distaste and has over time evolved to include other self-protection systems and boundaries [13], [14]. Subsequently, disgust is considered a basic response to a wide range of stimuli that may signal unhygienic contamination and the potential for disease [13]. Therefore, we decided to include behavioural tasks consisting of stimuli from the four disgust subtypes for more complete coverage of this basic emotion: core disgust (e.g., eat a biscuit with a living worm on it), moral disgust (e.g., put on a shirt of a paedophile, worn during sexual acts), animal-reminder disgust (e.g., hold the bone in your hands of a dead animal) and contamination disgust (e.g., place a used underpants/knickers in a laundry bag) [15]. We measured participants’ subjective and behavioural responses in the context of these four subtypes of disgust.

In order to test if sexual arousal attenuates the disgust properties of particular stimuli, we used an erotic film to induce sexual arousal. To control for the influence of mere positive arousal we also included a more generally arousing film clip (positive arousal), whereas a neutral film clip was added in order to serve as the baseline condition.

## Method

### Participants

Healthy female students (*n = *90, mean age = 23.12; SD = 1.99) were recruited at the University of Groningen via advertisement on university premises. The experiment was advertised as a study on ‘arousing films and behavioural tasks’ and no mention of either disgust or sex was made so as to minimize selection-bias. Screening was conducted with all participants in order to include only participants who had no sexual dysfunctions as the presence of sexual problems might affect participants’ responding. All participants reported moderate alcohol and nicotine consumption at most, and all denied hard drug use. All participants in this study were exclusively heterosexual. There was no significant difference between the three groups (*p*>.08) on several socio-demographic data (e.g., mood complaints, age, education, relationship status, last sexual contact, and contraception use).

We asked potential participants to come for testing in the laboratory on a date that they could select from our internal university system that is regularly used for student recruitment at our university. We provided the participants with the standardized information about the nature of the study. Every potential individual wanted to participate in the study after they read the information. Then we randomly allocated every participant in one of the following 3 groups: a sexually aroused, a positively aroused and a neutral group. Each of the three groups consisted of 30 participants.

### Mood Induction Stimuli Material

The mood-induction stimuli consisted of 3 films that were used in a between subjects design: i) a female friendly erotica ("de Gast"" by Christine le Duc) that was selected to induce sexual arousal; ii) a sports/high-adrenalin arousal clip (e.g., rafting/sky diving/mountain climbing) that served to induce arousal to control for general type of positive arousal; and iii) a neutral film consisting of a train-ride exposed to different sceneries, as a baseline or reference condition. Each film clip had a duration of 35 minutes. The latter two film clips were selected by the research team themselves from a selection of publically available film clips. Each film clip was validated and pilot tested with a group of 15 female students who did not participate in the actual study. The three films selected were successful in eliciting the intended affective state, Table 1. These students watched the 3 selected films and were asked to rate on Visual Analogue Scales (VAS) with a length of 10 cm, how much they feel the film was eliciting a feeling of general (positive) arousal, and sexual arousal ranging from zero = not at all to 10 = very. Table 1, illustrates the subjective evaluation of each stimulus-type on the dimensions of general arousal and sexual arousal. The general pattern of subjective ratings attests to the validity of the stimulus materials, Table 1. To examine in more detail whether the selected film material were able to elicit the intended emotion, we evaluated the relevant comparisons by means of t–tests, Table 1.

**Table 1:** Subjective evaluation for each dimension as a function of stimulus type.

*Emotion*	Erotic	Positive arousal	Neutral
*General arousal*	4.3 (1.9)^a, x^	8.5 (1.7)^b, y^	0.1 (0.4)^c^
*Sexual arousal*	9.4 (1.2)^b, z^	2.1 (1.6)^c, x^	0.2 (0.4)^c^

M(SD) M, mean, SD, standard deviation. Stimulus type includes the three film categories (erotic, positive arousal and neutral film) and the dimension includes the subjective elicited mood (sexual arousal, and general arousal). Different letters in superscript (a/b/c/d) indicate significant difference between stimulus categories within a dimension (***p*** **<.025**). For instance, the ‘a’ on the erotic and the ‘b’ on positive arousing film clip on the first row indicates that they do differ significantly from each other on the dimension of general arousal. The 2^nd^ letter (x) applies to relevant comparisons across columns. For instance the ‘x’ of the erotic film clip, on the dimension of sexual arousal with the ‘x’ on positive arousing film on the dimension of general arousal indicates that these two do not differ significantly from each other (***p*** **>.025**).

### Behavioural Tasks

We had 16 behavioural tasks/cues that participants were asked to conduct the requested assignment on, 4 tasks per each relevant disgust type. As mentioned in the introduction we used 4 different disgust types, namely, core, contamination, animal-reminder, and moral disgust. Appendix S1 provides a detailed description of the 16 behavioural tasks. The subcategory of core disgust included the tasks as numbered in the Appendix S1 that is 1, 2, 3, 4; moral disgust included task number 5, 6, 7, 8; animal-reminder disgust included task numbers 9, 10, 11, 12; and contamination disgust included tasks number 13, 14, 15, 16. Part of these behavioural tasks was composed of sex related stimuli or stimuli referring directly to sex, including task numbers 5, 8, 11, 15, 16. The latter two categories were initially decided on, by the research team, which was composed of a PhD student, three Master’s students and a psychology professor. In addition we (post hoc) invited 20 psychology students, independent of our sample to rate the stimuli (i.e., 16 behavioural tasks) on the dimension of sex relevance. The ratings were done on VAS that ranged from zero = not relevant at all to 100 = highly relevant. We included two other dimensions (i.e., food relevant and contamination relevant) to make the main aim less obvious for participants. By and large these data confirmed our a priori division, in terms of sex relevance. The mean score of the sex relevant tasks (M = 67.5, SD = 9.8) differed significantly from the mean score of the non-sex relevant items (M = 8.6, SD = 3.1), *t*(19) = 22.9, *p*<.001, on sex relevance. The median was 8.7 and scores ranged from 1.1 to 41.3 for the non-sex relevant tasks, and for the sex relevant tasks the median was 69.6, and scores ranged from 46.4 to 83.9, respectively. These descriptive statistics support the validity of the a priori assignment to sex vs. non-sex category. Yet, it also shows that Task 7 differed considerably from the other items in the group of non-sex relevant, in that it was rated relatively high on sex relevance (M = 41.3). Therefore, we decided to run the analysis with and without Task 7. On the whole this produced the same pattern of results. Based on the discussions and attention the research team invested in selecting disgusting sex relevant and non-sex relevant tasks, and because the results did not change, we decided to retain the a priori division in categories, thus leaving Task 7 (i.e., to come in contact with a shirt worn by a paedophile) in the non-sex relevant (moral) category. For details see Appendix S3. The authors are willing to share the additional analysis with interested readers. Please contact first author for such requests.

Each task consisted of four steps given by the experimenter over a speaker: i) observe the task; ii) rate the impression of the task; iii) conduct the task; and as a final step, iv) rate the task after completion. As an index of reliability, we computed Cronbach’s alpha based on the subjective elicited disgust as measured by VAS, step 1. Cronbach’s alpha for non sex related stimuli was.85; and for sex related stimuli.76 thus the reliability of both scales in terms of internal consistency was satisfactory; additionally we calculated Cronbach’s alpha for the 4 disgust subtypes: core disgust stimuli.76; animal-reminder disgust stimuli.74; moral disgust stimuli.53; and for contamination disgust subtype.75. Thus, it can be concluded that the reliability of the various tasks used in this study is satisfactory, with only moral stimuli having low internal consistency.

## Measures

### Disgust Propensity and Sensitivity Scale Revised (DPSS-R)

The DPSS-R is a 16 item questionnaire that consists of two validated subscales that measure trait disgust propensity (i.e., tendency to respond with disgust to potential disgust elicitors) and trait disgust sensitivity (i.e., appraisal of experiencing disgust) [16]. Participants read sixteen propositions on the frequency of experiencing bodily sensations related to disgust (e.g., ‘‘Disgusting things make my stomach turn” for propensity, and ‘‘I think feeling disgust is bad for me, It scares me when I feel like fainting” for sensitivity), and indicated which best applied to them on a scale from 1 = never to 5 = always. The DPSS-R has been validated and used in a number of studies [16] and it is the first index that measures disgust propensity and disgust sensitivity irrespective of disgust elicitors [17]. The scale has been shown to be internally consistent [16] and has shown predictive validity for experiencing disgust in disgust-eliciting experimental tasks across all relevant disgust domains [18]. In previous studies the scale was shown to be reliable, with the DPSS-R and its subscales’ internal consistency all above Cronbach’s alpha of.78 [18], [19]. In our sample, the Cronbach’s alpha for disgust sensitivity was.72 and.75 for disgust propensity.

### Emotional Subjective Ratings

Participants were given two sheets with Visual Analogue Scales (VASs): to measure the impression of the task (step 1) and another for after the task was completed, step 4. The VAS was intended to rate their evaluation of their current mood e.g., how disgusted are you feeling at this moment? The participants had to mark with a pen on a VAS that ranged from zero = not at all to 10 = very. As a measure of the affect induced by the film clips (manipulation check), we also included a VAS to measure their feeling of sexual arousal. Additionally, the participants had to indicate using a binary score whether they indeed completed or decided not to do the task, with a zero = not done or 1 = completed.

## Procedure

The experiment took place in a quiet room, divided from the experimenter’s room by a one-way screen. Participants were seated in front of a large projection screen (1.5×1.5 metre) and had a table in front of them to conduct the tasks on. The experimenter was on the other side of the room behind a one-way divider, from where it was possible to observe the participant whilst giving instructions over a microphone, steps 1–4. Participants were warned before starting the experiment that they might be asked to view erotic images and that they would be asked to touch or do things that they could find unpleasant. They were told that they could decide not to conduct step 3 (the actual doing/approaching part) of the task and then to report whether they did conduct or if they declined. In the case of no task completion (i.e., not completing step 3), the participant was asked to imagine as if they actually did conduct the task requested and rate the emotions elicited. No participant opted to withdraw from the study once the explanation was given.

The design of the study entailed that participants had to watch a 5 minute film to set the mood. Next, the screen was set to freeze, and the experimenter brought in one stimulus. After two tasks (i.e., one stimulus at a time), the film continued for 2 minutes before the screen was set to freeze and the 2 subsequent tasks/stimuli were presented and so on, until they had completed the full set of 16 behavioural tasks. The 8 steps (4 steps for each stimulus) of the behavioural task had to be completed whilst the film was stopped and screen frozen. With each task, participants were handed a two loose-leaf rating sheet (one for rating at the impression of the task – step 1 and another for the rating after the task was completed – step 4) for each of the 16 tasks. The 16 tasks were counterbalanced: specifically we had 4 different orders for counter-balance. Each rating sheet was given a number that varied by the condition and the group/order they had been randomly allocated to. After the behavioural measures were completed participants were given a set of questionnaires to complete in private. Finally, participants were fully debriefed about the purpose of the experiment, the stimuli and the nature of the behavioural tasks. Appendix S1 illustrates the behavioural tasks as perceived by participants, and what the stimulus entailed in reality.

Refreshments were given to participants together with a modest monetary gift i.e., 10 Euros. The full duration of the experiment took 2 hours per participant. This study was approved by the University of Groningen Psychology Ethical Committee, ECP (ECP-code 10336-NE). Furthermore, written informed consent was obtained from all participants involved in the study.

## Results

### Manipulation Check of Induced Sexual Arousal as the Mood of Interest

As a manipulation check of affect induced per group, we conducted a one-way analysis of variance (ANOVA) to assess the impact of sexual arousal as the induced mood of interest, on group (sexual arousal, positive arousal and neutral/baseline) at the impression of the task presented, Step 1. That is to assess whether the mood induced was effective throughout the 16 tasks that had to be completed (step 1 of each task). There was a significant difference between the 3 groups on sexual arousal ratings *F*(2, 87) = 12.71, *p*<.01. Attesting to the validity of mood induction, post hoc comparisons using LSD tests indicated that the sexual arousal group expressed significantly higher scores on sexual arousal (M = 1.4, SD = 1.0), compared to the neutral group (M = .53, SD = .82, *p*<.01) and the positive arousal group (M = .40, SD = .59, *p*<.01).

### Propensity and Sensitivity Disgust Traits as Measured by the DPSS-R

To verify the comparability of the three groups with regard to trait disgust sensitivity (DPSS-Sensitivity) or/and trait disgust propensity (DPSS-Propensity), we conducted a between group ANOVA on these variables. Supporting an equal distribution of scores on these disgust personality traits across groups, there were no significant differences between the 3 groups on trait disgust sensitivity *F*(2, 87) = 1.79, *p = *.2, η = .04 or trait disgust propensity *F*(2, 87) = .95, *p*>.4, η = .02. The means on the DPSS-Sensitivity were 9.2, 8.9, and 10.8; whereas on the DPSS-Propensity the means were 16.6, 16.3, and 15.4, for the sexual arousal, the positive arousal and the neutral group, respectively.

### The Influence of Sexual Arousal on Elicited Feelings of Disgust with Disgusting Sex versus Non-sex Related Stimuli

A mixed ANOVA, with 3 group (sexual arousal, positive arousal and neutral) as between-subject factor×2 type (sex related vs. non-sex related disgusting task) as within-subject factor, was conducted to assess the impact of the mood induction on the perception of disgust on sex and non-sex related disgusting tasks. There was a main effect of group *F*(2, 87) = 4.52, *p*<.01, η = .09 and a main effect of stimulus type *F*(1, 87) = 4.98, *p*<.05, η = .05. Yet, these main effects were qualified by a significant interaction of stimulus type * group *F*(2, 87) = 4.63, *p*<.01, η = .10.

To further examine this interaction term, we conducted two one-way ANOVA’s comparing the three groups on disgust ratings for both sex related disgusting tasks and non-sex related disgusting tasks. The first ANOVA with ratings for the sex related stimuli showed significant difference between groups *F*(2, 87) = 6.35, *p*<.01. Thus we conducted post hoc comparisons using LSD tests which indicated that the participants in the sexual arousal group rated the sex related stimuli significantly less disgusting than the positive arousal group (M-diff = −1.22, SD = .44, *p*<.01) and also less disgusting than the neutral group (M-diff = −1.47, SD = .44, *p*<.01). There was no meaningful difference between the positive arousal and the neutral group (*p = *.58). In the second ANOVA with the non-sex related stimuli, the global pattern was very similar although the group difference did not reach the conventional level of statistical significance *F*(2, 87) = 2.86, *p = *.06. Yet, paired comparisons using LSD tests indicated that the participants in the sexual arousal group rated the non-sex stimuli as less disgusting than the neutral control group (M-diff = −1.06, SD = .46, *p*<.05). As illustrated in Table 2, the difference between sexual arousal and positive arousal group did not reach significance (*p = *.57) and neither did the difference between the positive arousal and the neutral control group (*p = *.08). Appendix S2 demonstrates the means of the subjective disgust ratings for each of the 16 behavioural tasks per group, and shows that the pattern of findings was highly consistent across all tasks.

**Table 2:** Perceived level of elicited disgust as a function of group, stimulus type and time of measurement (before vs. after task).

	Sex related stimuli		Non-sex related stimuli	
Group	*before task*	*after task*	*before task*	*after task*
Neutral	6.9 (1.4)	6.8 (1.8)	6.6 (1.3)	7.1 (1.5)
Sexual arousal	5.4 (1.9)	5.7 (1.8)	5.6 (1.9)	6.1 (1.5)
Positive arousal	6.6 (1.8)	6.8 (2.1)	5.8 (2.1)	6.7 (2.9)

M(SD) M, means and (SD) standard deviations of the elicited disgust measured on a VAS per group.

### The Influence of Sexual Arousal on Elicited Feelings of Disgust from Differential Disgust Subtypes

A mixed ANOVA, with 3 group (sexual arousal, positive arousal and neutral) as between-subject factor×4 type (core, animal-reminder, contamination and moral disgust) as within-subject factor, was conducted to assess the impact of mood induction on the feelings of disgust elicited from the four different disgust subtypes. There was a significant effect of group *F*(2, 87) = 3.34, *p*<.05, η = .07 and a main effect of disgust type *F*(3, 85) = 49.64, *p*<.01, η = .36. However, there was no significant interaction of type*group *F*(6, 172) = 1.0, *p = *42, η = .02 hence, this effect of group was similar for all of the disgust subtypes. The pattern of the means for the 4 subtypes indicated that animal-reminder disgust elicited the highest disgust ratings, followed by core, contamination and moral disgust stimuli as shown in Table 3.

**Table 3:** Impact of sexual arousal on elicited feelings of disgust per disgust subtypes.

	Disgust subtypes			
Group	*Core*	*Moral*	*Animal-* *reminder*	*Contamination*
Neutral	7.4 (1.9)	5.2 (1.7)	7.7 (1.5)	6.2 (1.6)
Sexualarousal	6.0 (2.3)	4.4 (1.7)	6.4 (2.1)	5.2 (2.2)
Positivearousal	6.6 (2.3)	5.2 (1.8)	6.7 (2.3)	5.9 (2.2)
Totalscore	6.7 (2.2)	4.9 (1.8)	6.9 (2.0)	5.8 (2.1)

M(SD) M, means and (SD) standard deviations of elicited disgust per subtype as a function of group as measured on a VAS. Total score is the mean of the 3 groups per each disgust subtype.

### The Impact of Sexual Arousal on Actual Approach Behaviour and Task Performance

Here, we conducted a repeated measure ANOVA with 3 group (sexual arousal vs. positive arousal vs. neutral)×2 type (sex related vs. non-sex related disgusting tasks) on percentage of task completed. There was no significant interaction between type*group, Wilks **λ** = .98, *F*(2, 87) = .79, *p = *.46, η = .02. There was neither a main effect of task type Wilks **λ** = .97, *F*(1, 87) = 2.10, *p = *.15, η = .02. However, there was a substantial main effect of group *F*(2, 87) = 7.71, *p*<.01, η = .15. In line with predictions, paired comparisons using LSD tests revealed that the sexual arousal group conducted significantly more tasks than the neutral group (M-diff = 16.76, SD = 5.76, *p*<.01) and the positive arousal group (M-diff = 21.53, SD = 5.76, *p*<.01). The positive arousal group did not differ from the neutral group (M-diff = −4.77, SD = 5.76, *p*>.05). In line with our hypothesis both for the sex related disgusting tasks and for the non-sex related tasks, the sexual arousal group conducted the highest percentage of tasks compared to the other two groups. For the sex related tasks the means were 89.33%, 65.33%, and 74.01% for the sexual arousal, positive arousal and neutral group, respectively. Similarly, for the non-sex related tasks the means of task performed were 84.95%, 65.90%, and 66.77% for the sexual arousal, positive arousal and neutral group respectively.

### Sexual Arousal Modulates the Reduction in Disgust Following Task Performance

To test whether induced sexual arousal additionally modulates the reduction in feelings of disgust following the actual task performance, we conducted a 3 group (sexual arousal, positive arousal, neutral)×2 type (sex related vs. non-sex related tasks) ×2 time (pre task performance, post task performance) mixed ANOVA on elicited disgust. A main effect of time was noted *F*(1, 87) = 10.6, *p*<.01, η = .11 indicating that overall there was an increase in elicited disgust from pre to post task performance. However there was no time*group interaction *F*(1, 87) = .71, *p = *.49, η = .02. Therefore, this effect was found to be similar for all of the three groups, with no evidence to suggest that sexual arousal generally lessens feelings of disgust following task performance. Additionally, the effect of time varied across both task types *F*(1, 87) = 7.35, *p*<.01, η = .08. This indicated that overall the increase of disgust from pre to post task performance was strongest for the non-sex disgusting stimuli *t*(89) = 3.81, *p*<.001, η = .02. None of the other main and interaction effects, including the 3-way interaction between group, stimulus type and time reached significance. This pattern of results did not support the initial view, namely, that the reduction in disgust would be strongest for the sexual arousal group.

### A Test of Mediation

**Figure 1:**
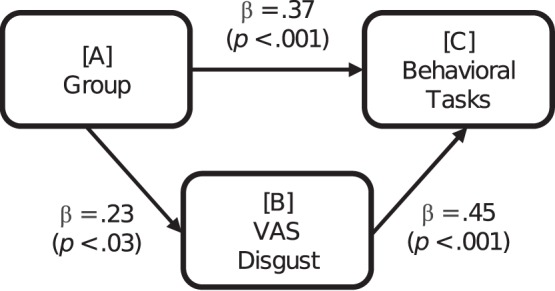
Testing mediation effects of self-reported disgust. Legend, [A] illustrates the experimental manipulation (sexual arousal group, versus both neutral and positive arousal group); [C] represents the Behavioural tasks and [B] show the subjective disgust as measured on the visual analogue scale (VAS); β is the beta value and *p* is the statistical significance level.

To test if the impact of the experimental manipulation (A, sexual arousal group, versus both neutral and positive arousal group) on approach behaviour during the actual behavioural task (C, Behavioural task), is mediated by changes in subjective disgust (B, VAS-disgust) we conducted 3 linear regression analysis for assumption checking (A>C, A>B, B>C), then we conducted a multiple regression analysis with (A, B>C) to test the mediation effect of (B). As illustrated in Figure 1, there was a trend for partial mediation with (B) still making a unique significant contribution, (β = .28, *p*<.005) also when both (A and B) were included in the equation. Thus the impact of induced sexual arousal on approach behaviour was not fully mediated by the influence of sexual arousal on subjective disgust. Hence, the change in approach behaviour and the change in subjective disgust seem largely independent outcomes of the induced sexual arousal.

### Influence Manipulation as a Function of Trait Disgust

Finally we explored, whether the effect of the sexual arousal induction might have varied according to the level of self-reported disgust susceptibility (i.e., disgust propensity). We conducted two linear regressions, the first analysis to predict the subjective elicited disgust and the second analysis for the prediction of percentage of behavioural tasks completed. We included Group, and DPSS-Propensity disgust trait at first level and in the second level we included the interaction term (Group*Disgust trait). In line with expectations the first analysis showed that the main effect of DPSS-Propensity reached the conventional level of significance (β = .40, *p* = .02). In the second step the DPSS-propensity retained significance whilst the interaction term (Group*Disgust trait) did not contribute significantly to the model (*p* = .49). Thus in line with predictions, independent of the film manipulation, high trait disgust participants responded generally with more disgust during the presented tasks. Similarly, we conducted the second regression analysis to test the influence of trait disgust (i.e., DPSS-propensity) on approach behaviour. At the first step the DPSS-Propensity reached the conventional level of significance (β = −4.9, *p* = .04) whilst in the second step the interaction term Group*Disgust trait did not approach significance (*p* = .11). This finding indicates that high disgust trait participants indeed completed less behavioural tasks.

## Discussion

The core findings can be summarized as follows: first, the sexual arousal group rated the sex related disgusting stimuli as significantly less disgusting when compared both to the neutral group and to the positive arousal group. A similar (non-significant) trend was evident for the non-sex related stimuli. Second, for both the sex and non-sex related disgusting tasks, the sexual arousal group conducted the highest percentage of tasks, indicating that sexual arousal indeed accentuates the *actual* approach tendency towards disgusting stimuli.

In line with predictions, when specifically considering the sexual arousal group, this group showed reduced elicited disgust towards the sex related (and to a certain extent also for the non-sex related) disgusting stimuli. This effect of sexual arousal on disgust cannot be attributed solely to positive arousal, given that the effects, especially at the behavioural level, were restricted to the sexual arousal condition. These results are congruent with the findings of a previous study conducted with male participants [6]. Although in the previous study the effects were restricted to disgust stimuli that referred directly to sex, in the present study the effect of induced sexual arousal was also evident for stimuli that do not directly refer to sex, Appendix S2. This apparent difference between studies could perhaps be attributed to the intensity of the experimental manipulation as Stevenson and colleagues used slides instead of a film clip to elicit sexual arousal [6].

The current study presents evidence that, similar to men, sexual arousal in women attenuates the elicited disgust of particular disgusting stimuli [6]. Importantly, however, our findings go further than merely replicating the self-report data of the aforementioned studies through demonstrating that sexual arousal also affects participants’ behaviour and attenuates actual approach tendencies. This seems particularly relevant here, when one considers that the subjective self-reported disgust does not mediate the impact of the experimental condition on the willingness to approach and conduct the tasks. This suggests that sexual arousal seems to have a largely independent influence on the experience of disgust and on people’s tendency to avoid disgust-relevant stimuli.

Although, participants in the sexual arousal group rated the non-sex relevant stimuli as less disgusting than the neutral control group, such difference was absent between the sexual arousal- and the positive arousal group. This could indicate that the impact of the sex film on subjective disgust is mainly driven by the generally arousing properties of the same sex film. Thus, the impact of the sex film on the subjective appreciation of sex relevant disgust elicitors might be driven by its specific power to elicit sexual arousal, whereas its effect on the appreciation of non-sex disgust elicitors might be more driven by its generally (sex independent) arousing properties. The impact of the sex film on participants’ actual approach of sex relevant and sex irrelevant disgust elicitors seems specifically driven by its power to elicit sexual arousal, as the sex irrelevant arousing films did not affect participants’ avoidance tendencies (neither for the non-sex nor for the sex relevant disgusting tasks). Together the present pattern of findings not only shows that feelings and avoidance of disgust represent (partly) independent phenomena, it also suggests that they are differentially influenced by sexual arousal. Perhaps most important for the present context, the findings indicate that both the impact of heightened sexual arousal on subjective disgust and also on disgust-induced avoidance will act in a way to facilitate the engagement in pleasurable sex and can be problematic if one of the two is not influenced or modified by sexual arousal.

From a clinical standpoint these findings can indicate that lack of sexual arousal (perhaps due to inappropriate stimulation) may interfere with functional sex, as it may prevent the reduction of disgust and disgust related avoidance tendencies. Consequently, if sexual arousal is low (for a variety of possible reasons), the disgusting properties of specific stimuli, which are relevant for the engagement in pleasurable sex, as well as the hesitation to approach these stimuli are not attenuated. As a result, this could lead to problems with sexual engagement, and lack of vaginal lubrication, which in turn could increase friction and cause problems such as pain with intercourse. It is thus possible that in extreme cases the woman might acquire negative associations with sex and might start to avoid sexual intercourse altogether. Relevant to this, our previous studies with women suffering from *vaginismus* (Genito-pelvic pain disorder/penetration disorder) have shown that they experience disgust responses towards erotic stimulation at the subjective as well as at a more automatic level [4], [5]. Moreover, the fact that sex related stimuli appeared to elicit disgust rather than arousal in women suffering from vaginismus might further worsen the problem. This is relevant here, since a typical response to disgust is avoidance behaviour in order to create distance from the disgusting stimuli. Thus, it is highly possible that these sexual problems can be directly or indirectly related to low sexual arousal, which as a consequence gives more room for the elicitation of disgust, resulting in a downward spiral and continued maintenance of their difficulties and sexual dysfunction.

Sexual-arousal-induced reduction of people’s avoidance of disgust-relevant stimuli was not restricted to sexual stimuli but seems to reflect a more general phenomenon that also applies to disgusting stimuli in general. The result that sexual arousal was quite similar across various categories further underlines the conclusion that the influence of sexual arousal reflects a more general phenomenon (not restricted to sex related disgust stimuli or any other subtype of disgust).

The absence of a decrease of (sexual) disgust after actual exposure to the disgusting tasks (following sexual arousal induction) could indicate that there was no additional impact on the rate of habituation. However, it should be noted that due to the weakening influence of sexual arousal on the initial feelings of disgust at the starting point, there was already a difference between conditions, leaving less room for further reduction in the sexual arousal group.

### Limitations and Further Studies

Some limitations should be mentioned: to verify the efficacy of our experimental manipulation we have entirely relied on subjective ratings of participants’ sexual arousal; it would be interesting to see whether this film clip is also successful in eliciting physiological arousal in addition to subjective sexual arousal. A physiological measure (e.g., vaginal photoplethysmograph) would be appropriate because strictly speaking, in the current design it cannot be ruled out that test- and experimenter demands might have played a role in participants’ ratings of the manipulation check question about their sexual arousal. However, this may be considered unlikely, as the fact that, at the behavioural level specifically the sex arousal group showed less avoidance behaviour would be inconsistent with a demand explanation.

Furthermore, although this study refers to sex related disgusting tasks and to non-sex related disgusting tasks, we cannot be entirely sure, if what we denote as sex related actually differed from the non-sex related disgusting stimuli in the perception of the current participants in terms of sexual relevance (vs. non-sex relevant). Yet, by and large the ratings of an independent group of participants confirmed the validity of the present division in a sex relevant versus a non-sex relevant category. Although it should still be acknowledged that the task referring to a shirt worn by a paedophile clearly diverged in terms of reported sex relevance from the other stimuli (that were a priori assigned to the non-sex category). Therefore, we re-ran the analyses without this particular task. Removing this task had no meaningful impact on the outcome of the analyses. This renders it unlikely that the absence of a differential impact of sexual arousal on sex relevant versus non-sex relevant stimuli could be attributed to flaws in the categorisation of our tasks, thereby sustaining the validity of the current pattern of findings.

Automatic avoidance tendencies might be critically involved in the affective, behavioural and physiological processes relevant for sexual engagement. Thus, it would be important to further investigate whether the findings of this study are also evident for the more automatic, reflexive physiological disgust response that can be assessed using an electromyography (EMG) of the *levator labii* [4] or the pelvic floor muscles [20] as relatively uncontrollable defensive responses.

In addition, it would be interesting to investigate the influence of sexual arousal on the disgust eliciting properties of particular stimuli in different groups. Perhaps in women with sexual dysfunction such as dyspareunia or vaginismus, arousal does not impact on disgust which might help explain the occurrence and persistence of sexual pain or vaginistic symptoms.

### Conclusions

The current findings enhance our understanding of how sexual arousal interplays with disgust and disgust eliciting properties of both sex and non sex related disgusting stimuli in women. Specifically, these findings further the existing literature-base by showing that this relationship goes beyond subjective reports to reach the behavioural level through facilitating the actual approach to the same stimuli. In other words, this study might help develop our insight into the quandary as to why people still manage to engage in pleasurable sex despite the disgusting nature of many stimuli that are implicated in sexual behaviours. The present array of findings not only suggests that high sexual arousal may facilitate common sexual behaviours but also suggests that low sexual arousal might be a key feature in the maintenance of particular sexual problems or dysfunctions.

## Supporting Information

Appendix S1 **These behavioural tasks were given randomized in a set of 2, each time following 2 minutes film clip.** Each task was given in 4 steps (*See* Method).
(DOC)

Appendix S2 **Means and (SD) standard deviations of the subjective disgust ratings for each behavioural task per group in order to show that the pattern of findings seem to be similar for all of the 16 behavioural tasks.**
(DOC)

Appendix S3 **Means, and Standard Deviations (SD), of the subjective (post hoc) ratings for each of the 16 behavioural tasks.** The sex relevance is the mean result from the VAS. Task number 5, 8, 11, 15 and 16 are the behavioural tasks considered sex relevant.
(DOC)
