# Supplementary material for: Feelings of Disgust and Disgust-Induced Avoidance Weaken following Induced Sexual Arousal in Women
Source: PLoS One. 2012 Sep 12;7(9):e44111. doi: 10.1371/journal.pone.0044111 (PMC3440388; doi:10.1371/journal.pone.0044111)
Supplement: Appendix S1 — These behavioural tasks were given randomized in a set of 2, each time following 2 minutes film clip. Each task was given in 4 steps (See Method). (DOC) [file pone.0044111.s001.doc]

**Appendix-S1.** Behavioural tasks as perceived by participants

|  | As Perceived | In Reality |
| --- | --- | --- |
| 1 | take a sip of the juice with a large insect in the plastic cup | *insect was made of plastic* |
| 2 | remove the used toilet paper from the jar and put it back in place | *toilet paper was spoiled with sweet bread to give the impression of faeces* |
| 3 | wipe your hands with the used tissue | *tissue was touched with yellow-brownish ink* |
| 4 | take a bite from the biscuit, which is lying next to a living worm | *the worm was indeed living and was kept for the duration of the experiments in a large container with blocks of soil in it and it was taken back to the same field when experiments were completed* |
| 5 | lubricate the vibrator with your hands | *the vibrator was clean* |
| 6 | insert the needle into the heart of the voodoo doll representing the person you hate | *n/a* |
| 7 | this shirt belongs to a paedophile that was used during rape - take the shirt out of the bag and hug it | *the shirt was new and clean* |
| 8 | read the story and say aloud: “It was so horny to have him (the dog) inside me” | *n/a* |
| 9 | touch the (unattached) wet human hair | *the hair belonged to one of the researchers and it was wet with a neutral lubricant* |
| 10 | hold the bone for 5 seconds | *the bone was a ‘dog’s chewing bone’ lubricated with red ink* |
| 11 | discard the used women tampon | *the tampon was new that has socked in red ink and water* |
| 12 | stick a needle in the eye of a cow | *the eye of the cow was real, and participants had to only touch it with the needle provided – a new eye ball was brought every day as frozen from the butcher and taken back at the end of the day for proper biohazard waste removal* |
| 13 | hold a bandage that was used on a wound for 5 seconds | *the bandage was new and spoiled with red and brownish ink* |
| 14 | rub the used toothbrush back and forth on your cheek for five seconds | *the toothbrush was new* |
| 15 | place the used panties/knickers of a woman in a bag | *the panties were unused and spoiled with drops of coconut milk* |
| 16 | stick your finger in the bowl of used condoms and touch each one of them | *the condoms were new and wet with penile lubricant* |
