# Supplementary material for: Feelings of Disgust and Disgust-Induced Avoidance Weaken following Induced Sexual Arousal in Women
Source: PLoS One. 2012 Sep 12;7(9):e44111. doi: 10.1371/journal.pone.0044111 (PMC3440388; doi:10.1371/journal.pone.0044111)
Supplement: Appendix S2 — Means and (SD) standard deviations of the subjective disgust ratings for each behavioural task per group in order to show that the pattern of findings seem to be similar for all of the 16 behavioural tasks. (DOC) [file pone.0044111.s002.doc]

**Appendix-S2.** Subjective disgust per each behavioural task

| Group | Sexual arousal | | Positive arousal | | Neutral | |
| --- | --- | --- | --- | --- | --- | --- |
| *Elicited emotion* | *Disgust* | *Sexual arousal* | *Disgust* | *Sexual arousal* | *Disgust* | *Sexual arousal* |
|  | M (SD) | M (SD) | M (SD) | M (SD) | M (SD) | M (SD) |
| Task 1 | 4.4 (2.9) | 1.1 (1.3) | 4.8 (3.8) | 0.1 (0.2) | 6.3 (3.4) | 0.3 (0.7) |
| Task 2 | 6.7 (2.9) | 1.3 (1.4) | 7.1 (3.2) | 0.2 (0.5) | 7.7 (2.4) | 0.4 (1.1) |
| Task 3 | 6.2 (2.6) | 1.7 (1.9) | 7.2 (2.8) | 0.2 (0.3) | 7.1 (2.4) | 0.2 (0.6) |
| Task 4 | 6.5 (3.0) | 1.1 (1.5) | 7.3 (2.7) | 0.1 (0.2) | 8.5 (2.1) | 0.2 (0.5) |
| Task 5 | 2.4 (1.6) | 3.1 (1.9) | 3.7 (2.7) | 1.8 (2.3) | 3.5 (2.7) | 1.5 (1.5) |
| Task 6 | 2.6 (2.4) | 1.5 (1.6) | 2.7 (2.6) | 0.1 (0.2) | 2.8 (3.1) | 0.3 (0.9) |
| Task 7 | 5.4 (3.2) | 1.1 (1.2) | 5.7 (3.3) | 0.2 (0.6) | 5.8 (3.3) | 0.5 (0.9) |
| Task 8 | 7.0 (3.0) | 1.2 (1.5) | 8.5 (2.3) | 0.5 (0.9) | 8.7 (2.4) | 0.8 (1.4) |
| Task 9 | 6.1 (2.5) | 1.5 (1.6) | 6.2 (3.3) | 0.2 (0.3) | 6.6 (2.6) | 0.3 (0.6) |
| Task 10 | 5.6 (3.2) | 1.6 (1.8) | 5.8 (3.2) | 0.2 (0.4) | 7.4 (2.5) | 0.2 (0.6) |
| Task 11 | 6.3 (2.7) | 1.7 (1.9) | 7.9 (2.4) | 0.4 (0.8) | 8.2 (1.8) | 0.4 (1.0) |
| Task 12 | 7.6 (2.3) | 0.7 (0.9) | 7.1 (2.9) | 0.2 (0.4) | 8.6 (1.6) | 0.2 (0.6) |
| Task 13 | 5.1 (2.3) | 1.2 (1.3) | 5.7 (3.2) | 0.3 (0.7) | 6.2 (2.7) | 0.2 (0.7) |
| Task 14 | 4.5 (3.2) | 1.3 (1.4) | 4.9 (2.9) | 0.6 (1.8) | 4.7 (3.0) | 0.4 (1.0) |
| Task 15 | 5.2 (2.2) | 1.3 (1.4) | 6.1 (2.6) | 0.7 (1.8) | 6.9 (1.6) | 0.5 (1.3) |
| Task 16 | 6.2 (3.1) | 2.5 (2.1) | 7.0 (2.7) | 1.3 (2.0) | 7.1 (2.5) | 1.7 (2.4) |
