# Supplementary material for: Feelings of Disgust and Disgust-Induced Avoidance Weaken following Induced Sexual Arousal in Women
Source: PLoS One. 2012 Sep 12;7(9):e44111. doi: 10.1371/journal.pone.0044111 (PMC3440388; doi:10.1371/journal.pone.0044111)
Supplement: Appendix S3 — Means, and Standard Deviations (SD), of the subjective (post hoc) ratings for each of the 16 behavioural tasks. The sex relevance is the mean result from the VAS. Task number 5, 8, 11, 15 and 16 are the behavioural tasks considered sex relevant. (DOC) [file pone.0044111.s003.doc]

**Appendix-S3.** Sex relevance per each behavioural task.

|  | *Sex Relevance* |
| --- | --- |
|  | M (SD) |
| Task 1 | 7.7 (13.2) |
| Task 2 | 3.1 (5.1) |
| Task 3 | 8.5 (12.8) |
| Task 4 | 2.9 (3.1) |
| Task 5 | 83.9 (9.4) |
| Task 6 | 3.3 (5.8) |
| Task 7 | 41.3 (18.6) |
| Task 8 | 77.2 (7.7) |
| Task 9 | 15.0 (17.1) |
| Task 10 | 6.1 (6.6) |
| Task 11 | 46.4 (19.9) |
| Task 12 | 2.0 (3.0) |
| Task 13 | 5.1 (2.3) |
| Task 14 | 3.5 (4.7) |
| Task 15 | 54.6 (23.9) |
| Task 16 | 75.4 (12.7) |
